# Combinatorial stresses kill pathogenic *Candida* species

**DOI:** 10.3109/13693786.2012.672770

**Published:** 2012-04-02

**Authors:** Despoina Kaloriti, Anna Tillmann, Emily Cook, Mette Jacobsen, Tao You, Megan Lenardon, Lauren Ames, Mauricio Barahona, Komelapriya Chandrasekaran, George Coghill, Daniel Goodman, Neil A. R. Gow, Celso Grebogi, Hsueh-Lui Ho, Piers Ingram, Andrew McDonagh, Alessandro P. S. De Moura, Wei Pang, Melanie Puttnam, Elahe Radmaneshfar, Maria Carmen Romano, Daniel Silk, Jaroslav Stark, Michael Stumpf, Marco Thiel, Thomas Thorne, Jane Usher, Zhikang Yin, Ken Haynes, Alistair J. P. Brown

**Affiliations:** * School of Medical Sciences, University of Aberdeen, Institute of Medical Sciences, Aberdeen; † Department of Biosciences, College of Life and Environmental Sciences, University of Exeter, Exeter; ‡ Institute for Complex Systems and Mathematical Biology, School of Natural and Computing Sciences, University of Aberdeen, Aberdeen; § Department of Mathematics, Imperial College London; # School of Natural and Computing Sciences, University of Aberdeen, Aberdeen; ⁁ Department of Microbiology, Imperial College London, London; $ Centre for Bioinformatics, Division of Molecular Biosciences, Imperial College London; ° Formerly of the Department of Mathematics, Imperial College London, London, UK

**Keywords:** *Candida albicans*, *Candida glabrata*, osmotic stress, oxidative stress, nitrosative stress, combinatorial stress

## Abstract

Pathogenic microbes exist in dynamic niches and have evolved robust adaptive responses to promote survival in their hosts. The major fungal pathogens of humans, *Candida albicans* and *Candida glabrata*, are exposed to a range of environmental stresses in their hosts including osmotic, oxidative and nitrosative stresses. Significant efforts have been devoted to the characterization of the adaptive responses to each of these stresses. In the wild, cells are frequently exposed simultaneously to combinations of these stresses and yet the effects of such combinatorial stresses have not been explored. We have developed a common experimental platform to facilitate the comparison of combinatorial stress responses in *C. glabrata* and *C. albicans*. This platform is based on the growth of cells in buffered rich medium at 30°C, and was used to define relatively low, medium and high doses of osmotic (NaCl), oxidative (H _2_O_2_) and nitrosative stresses (e.g., dipropylenetriamine (DPTA)-NONOate). The effects of combinatorial stresses were compared with the corresponding individual stresses under these growth conditions. We show for the first time that certain combinations of combinatorial stress are especially potent in terms of their ability to kill *C. albicans* and *C. glabrata* and/or inhibit their growth. This was the case for combinations of osmotic plus oxidative stress and for oxidative plus nitrosative stress. We predict that combinatorial stresses may be highly signif cant in host defences against these pathogenic yeasts.

## Introduction

Some pathogenic *Candida* species are commensal microbial flora of the gut and vagina [1,2]. However in immunocompromised patients, these organisms can disseminate through the bloodstream, colonize internal organs and cause life-threatening systemic *candidiasis* [1,2]. Indeed, *Candida* is responsible for one-fourth of hospital-acquired infections [3]. *Candida albicans* is the most frequently isolated species from such infections, and *Candida glabrata* is the second most common species [4].

A range of clinically useful antifungal drugs is available to combat such infections, including polyenes, azoles and echinocandins [5]. However, significant efforts are being devoted to the development of new antifungal therapies because resistance to the current antifungal drugs is arising [6], and some drugs exert side-effects such as nephrotoxicity [7]. In principle, an increased understanding of how pathogenic *Candida* species adapt and survive in their host could facilitate the design of new therapeutic strategies.

All microorganisms must adapt to dynamic environmental challenges if they are to survive. In particular, the success of *C. albicans* and *C. glabrata* as pathogens is dependent upon their ability to adapt to the environmental stresses they encounter within the diverse niches they occupy in their human host [8]. For example, when *Candida* cells are engulfed by phagocytic cells, they are exposed to reactive oxygen species and reactive nitrogen species [9,10]. Host immune cells also activate intracellular ion currents [11] that might expose *Candida* cells to cationic and osmotic stresses. Responses to osmotic stress may also be relevant in oral infections and during colonization of skin and kidney tissues. The exact potency of osmotic insults that individual *Candida* cells face in their immediate microenvironments *in vivo* is not clear. Nevertheless, it is not surprising that both *C. albicans* and *C. glabrata* have evolved robust responses to cationic/osmotic, oxidative and nitrosative stresses [12–15].

The stress-activated protein kinase, Hog1, is activated upon osmotic, oxidative and acetate stress in the benign model yeast, *Saccharomyces cerevisiae* [16]. In *C. albicans*, Hog1 also plays a key role in the osmotic stress response and contributes to the oxidative stress response [17,18]. In *C. glabrata*, Hog1 seems to have similar function to ScHog1 and this MAP kinase is also required for resistance to weak acids such as sorbic acid [19]. However, the upstream signalling modules that activate Hog1 have diverged signif cantly between *C. albicans*, *C. glabrata* and *S. cerevisiae* [19,20].

The molecular responses of *S. cerevisiae* to oxidative stress are dependent on the transcription factor Yap1 [21,22]. Orthologues of Yap1 (Cap1) are present in both *C. albicans* and *C. glabrata* and they are required for the activation of the transcriptional response to reactive oxygen species and for resistance to this stress [8,23–25].

The regulation of nitrosative stress responses in yeasts has been less well studied. In *C. albicans*, the transcriptional response to reactive nitrogen species is dependent on the transcription factor Cta4 [14]. Cta4 activates nitrosa-tive stress genes such as *YHB1*, which is strongly induced in response to nitrosative stress and is required for the detoxification of reactive nitrogen species [13,26].

The transcription factors Msn2 and Msn4 also contribute to stress adaptation in *S. cerevisiae*. Msn2 and Msn4 activate a common set of genes that are induced in response to various environmental stresses –a phenomenon called the core or environmental stress response [27–30]. A similar situation exists in *C. glabrata* where Msn2 and Msn4 orthologues regulate the core transcriptional response to stress and share many common target genes with Msn2 and Msn4 in *S. cerevisiae* [31]. In contrast, in *C. albicans* the Msn2 orthologue, Mn11, does not play a role in the core stress response [18,32]. Instead, Mn11 regulates the response to weak acids in *C. albicans* [33].

Taken together, these and other data indicate that key stress regulatory modules have been conserved in these pathogenic and benign yeasts although *C. glabrata* and *C. albicans* are thought to have diverged from *S. cerevisiae* over 20 and 100 million years ago, respectively [e.g., 34,35]. However, in some cases the cellular roles of these regulatory modules have diverged, and upstream and downstream components on these signalling pathways are less well conserved, leading to the suggestion that fungal stress responses have evolved rapidly and in a niche-specif c manner [15].

These observations reflect the considerable efforts that have been made by many groups to elucidate the regulation of stress responses in model and in pathogenic yeasts. Almost without exception, these studies have examined responses to specific stresses in isolation with a view to excluding confounding factors. However, yeast cells are often exposed simultaneously to combinations of different stresses in their natural environments rather than to individual stresses in isolation. Therefore, it is important to examine microbial adaptation to such combinatorial stresses. We predict that the impact of combinatorial stresses is not simply equivalent to the additive effects of the corresponding individual stresses. We also predict that crosstalk between stress signalling pathways is likely, and that this crosstalk might exert antagonistic or cooperative effects upon particular stress responses. Such responses to the simultaneous exposure to combinatorial stresses will be distinct from the phenomenon of stress cross protection, where exposing yeast cells to one type of stress can protect these cells against a subsequent exposure to a different type of stress [18,36]. Therefore, our long-term aim is to test these predictions and to def ne the dynamic responses of medically relevant combinatorial stresses upon the major fungal pathogens, *C. glabrata* and *C. albicans*. However, to achieve this we have had to establish a common experimental platform that allows us to investigate combinatorial stress responses to immunologically important stresses in these divergent yeasts. In this paper, we describe our rationale behind the design of this experimental platform, the development of this platform, and its application to our initial analyses of combinatorial stresses in *C. albicans* and *C. glabrata*. We show for the first time that combinatorial stresses exert significant effects upon the growth of these pathogenic yeasts.

## Materials and methods

### Strains

The strains *C. albicans* NGY152, *C. albicans* SC5314 and *C. glabrata* ATCC2001 were used. The prototrophic *C. albicans* strain NGY152 [37] is CAI4 (*ura::λimm434/ura3::λimm434:* [38]) containing the *URA3* plasmid CIp10 [39], and this strain is derived from SC5314 [38]. *Candida albicans* NGY152 is congenic with the clinical isolate SC5314 [40]. *Candida glabrata* ATCC 2001 is a wild type reference strain obtained from the American Type Culture Collection (Manassas, VA, USA).

The *C. albicans* strain used for the Hog1-phosphorylation experiments was ML258 (*ura3::imm434/ura3:: imm434, RPSl/rpsl*::pACT1-FLAG-GFP) which is CAI-4 [38] with pACT1-FLAG-GFP integrated at the *RPS1* locus [41]. This strain expresses a FLAG-tagged version of GFP from the *ACT1* promoter, which functions as an internal control for Western blots.

### Growth conditions and stress induction

*Candida* cells were grown overnight at 30°C at 200 rpm in YPDT medium (Tris buffered YPD: 2% w/v glucose, 2% w/v mycological peptone, 1% w/v yeast extract, 100 mM Tzris.HCl, pH 7.4). On the day of the experiment, cultures were diluted in fresh pre-warmed YPDT to an OD_600_ of 0.2 and grown to an OD_600_ of 0.8 at 30°C at 200 rpm. Cells were then diluted four-fold in fresh pre-warmed YPDT, mixed with the appropriate stressor(s) at the specified concentration(s) and incubated at 30°C at 200 rpm for the specified period before analysis. Osmotic stress was imposed either with NaCl or sorbitol. Oxidative stress was applied using hydrogen peroxide (H_2_O_2_) or tert-butyl alcohol (tBOOH). Nitrosative stress was imposed using dipro-pylenetriamine (DPTA) or diethylenetriamine (DETA) NONOate.

### Cell growth and viability

Cell viability was determined following exposure to osmotic, oxidative and nitrosative stresses by propidium iodide staining and Fluorescence Activated Cell Sorting (FACSCalibur: Becton Dickinson, CA, USA). After exposure to stress, cells were harvested by centrifugation (4,000 rpm), washed and resuspended in modif ed FACS buffer (1×phosphate buffered saline (PBS), 2 mM ethylenedi-aminetetraacetic acid (EDTA), 0.5% bovine serum albumin (BSA), 0.01% Tween 20). Immediately before FACS analysis, 200μl of propidium iodide (2 μg/ml) was added to 500 μl of cell suspension containing approximately 10 ^7^ cells. For each condition 50,000 cells were analyzed through FACS, the procedures and data analyses being performed according to the manufacturer's instructions (Microbial Cytometry, BD FACSCalibur: BD Biosciences, San Jose, CA, USA) and following the methods described at Alberghina and co-workers [42].

Cell growth was monitored over 48 h in 96-well microplates at OD_620_ at 30°C using a FluoSTAR OPTIMA fluorometer and measurements were taken every 20 min. We quantified the length of the lag phase and the doubling time for each condition using the following approach. We regarded the lag phase as the time taken for cells to resume growth after exposure to stress. We further assumed that following this initial lag phase, cells resumed exponential growth, the rate of which was affected by nutrient availability and stress adaptation. Hence, the growth model was formulated as:


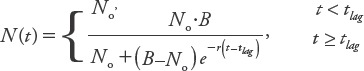


It has four parameters: *N*: initial population (i.e., initial optical density); *B*: carrying capacity (the stationary phase optical density); *r*: intrisinc growth parameter (the inverse of doubling time, with a dimension of h^−1^); and t_lag_: duration of lag phase (h).

All four parameters were optimized simultaneously using an evolutionary algorithm [43] that minimizes the sum of squares between a particular growth curve and corresponding model predictions. For each run, we used the following parameter values for the evolutionary algorithm: population size, 200 (this is the number of randomized individuals that the evolutionary algorithm creates, not the optical density of the cell); generation, 200; parent number, 30; pressure on fitness, 0.45; expected rate of convergence, 1. The four parameters were searched within their feasible ranges (*N_o_*:[0 1]; *B*: [0 2]; *r*: [0 1]; t^lag^: [0 48]). The value of the minimal sum of squares for each generation was tracked to guarantee that the evolutionary algorithm converges to a particular set of values.

### Western blotting

Whole cell lysates were prepared from stressed and unstressed control cells at various time points. Cells were resuspended in fresh protein lysis buffer (50 mM Tris-HCl pH 7.5, 150 mM NaCl, 0.5% NP40) containing inhibitors (2 mg/ml leupeptin, 2 mg/ml pepstatin, 1 mM phenylmeth-ylsulfonyl fluoride, 2 mM Na_3_VO_4_, 50 mM NaF) and lysed in a fast-prep machine. Protein extracts were clarified by centrifugation and protein concentration determined using a Bradford assay.

For *C. albicans*, 15 μg of total protein was loaded per lane of NuPAGE ® Novex Bis-Tris 4–12% sodium dodecyl sulfate (SDS)-polyacrylamide gels (Invitrogen, Paisley, UK). Proteins were transferred onto polyvinylidene difluoride (PVDF) membranes, blocked with 10% BSA in PBS containing 0.1% Tween 20 (PBS-T) for 30 min at room temperature and incubated overnight at 4°C with primary antibodies against phosphorylated Hog1 (anti-phospho-p38 MAPK (Thr180/Tyr182) XP Rabbit mAb, New England Biolabs, Herts, UK) and anti-FLAG antibody (Sigma-Aldrich, Dorset, UK). Blots were washed with PBS-T before incubation with secondary antibody (anti-rabbit IgG, HRP-linked antibody; New England Biolabs) for 1 h at room temperature. Blots were washed with PBS-T and developed using LumiGlo (New England Biolabs) according to the manufacturers’ instructions. Phosphorylated Hog1 and GFP-FLAG levels were visualized and quantified using the FluorChem FC2 (Alpha Innotech) system and the ratio of phosphorylated Hog1 to internal standard (GFP-FLAG) was calculated.

For *C. glabrata*, 15 μg of total protein were loaded per lane of 10% SDS-polyacrylamide gels. Gels were transferred onto PVDF membranes, blocked with 10% BSA in Tris-buffered saline containing 0.1% Tween (TBST) for 30 min at room temperature, and then incubated overnight at 4°C with primary antibody against phosphorylated p38 (Phospho-p38 MAPK (Thr180/Tyr182) XP Rabbit mAb: New England Biolabs, Ipswich, MA, USA). Blots were washed four times for 5 min at room temp with TBST, then incubated for 2 h at room temperature with anti-isotype HRP conjugated secondary antibody diluted in 5% BSA TBST. Blots were washed 3×5 min with TBST and developed using Lumiglo solution (New England Biolabs). To determine total Hog1 protein levels, blots were incubated in stripping buffer (0.7% β-mercaptoethanol, 2% SDS, 6.25% 1 M Tris-HCl pH 7) for 30 min at 50°C, washed six times for 10 min in TBST, then incubated for 30 min with labelled secondary antibody and developed as before to determine if previous antibody was no longer detectable. Blots were then washed again 3 × 10 min in TBST and incubated with primary anti-Hog1p antibody (sc-9079, Santa Cruz Biotechnology, Santa Cruz, CA, USA) overnight at 4°C in 5% BSA TBST. Blots were then washed, incubated with secondary antibody and developed as before. Phosphorylated and total Hog1 levels were then quantif ed using ImageJ software.

### Statistical analyses

All experiments were performed at least three times, and the data presented are the cumulative result of all experiments performed. Data are expressed as means plus standard deviations. SPSS for Windows version 19.0 was used for all statistical analyses. Associations between growth parameters, such as doubling time, lag phase, maximal biomass or propidium iodide staining were determined by one-way ANOVA and Dunnett *t*-tests. The unstressed samples were treated as controls and the values of all other samples were compared against the controls. The following *P*-values were considered: ^*^P<0.05; ^**^P<0.01; ^***^P<0.001.

### Results

#### Rationale behind the experimental platform

Our long-term goal is to compare the dynamic quantitative responses of *C. glabrata* and *C. albicans* to chemically diverse stresses at both the molecular and cellular levels. To achieve this goal, we required an experimental platform that allows direct comparison of the behaviors of these species under the chosen stress conditions.

Firstly, we needed to establish common growth conditions for *C. glabrata* and *C. albicans*. Most experimental analyses of stress responses in *C. glabrata* and *C. albicans* have been performed using rich YPD medium, and therefore we used this type of medium for our experiments. A growth temperature of 37°C is more relevant from a clinical perspective, but *C. albicans* undergoes yeast-hypha morphogenesis at this temperature [44]. Yeast and hyphal *C. albicans* cells display differing degrees of stress resistance and hence the induction of cellular morphogenesis during growth at 37°C would introduce confounding effects upon the dynamics of stress adaptation. Therefore, we examined cultures of yeast cells grown at 30°C to avoid these confounding effects.

Secondly, we used common growth conditions for all stresses of interest. Nitrosative stress responses are generally examined using buffered medium to ensure a uniform release of NO from chemical donors such as DPTA NON-Oate [13,45]. Therefore, we used Tris-buffered YPD at pH 7.4 (YPDT) for all experiments to avoid the confounding effects of differential medium pH upon our comparisons of different stressors.

Thirdly, we chose specific types of osmotic, oxidative and nitrosative stress for the majority of our work. Our choice of chemical stressors was based on their impact upon cell growth and viability, their chemical stability over the timescales of interest, their physiological relevance *in vivo* and their ability to activate the stress-regulated protein Hog1.

**Fig. 1:**
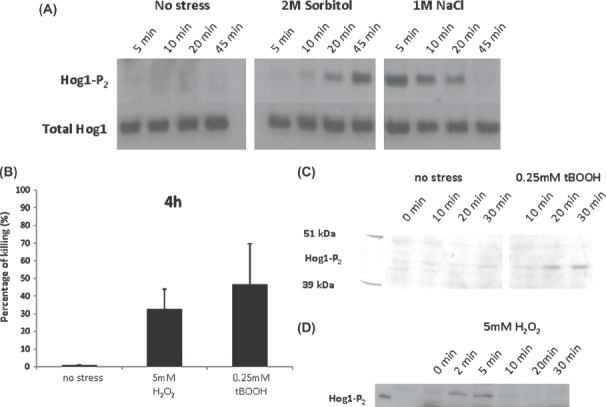
Comparison of the effects of specific stressors upon *Candida albicans* and *Candida glabrata* cells grown in YPDT at 30°C. (A) Impact of 2 M sorbitol and 1 M NaCl upon the dynamics of Hog1 phosphorylation in *C. glabrata*: upper panels, Western blots probed with a phosphospecific antibody against Hog1; lower panels, Western blots probed with an anti-Hog1 antibody that detects total Hog1 (loading control). (B) Impact of 4 h exposure to 5 mM H_2_O_2_ and 0.25 mM tBOOH upon *C. albicans* killing as quantif ed by propidium iodide staining and FACS analysis. (C) Dynamics of Hog1 phosphorylation in *C. albicans* following exposure to 0.25 mM tBOOH as assayed by Western blotting. (D) Dynamics of Hog1 phosphorylation in *C. albicans* following exposure to 5 mM H_2_O_2_.

Two types of osmotic stress were examined: sorbitol, which imposes osmotic stress alone, and NaCl, which imposes both cationic and osmotic stress. In *C. glabrata*, both NaCl and sorbitol induced Hog1 phosphorylation under the growth conditions examined, although there were differences in the dynamics of Hog1 phosphorylation (Fig. 1A). Hog1 phosphorylation was relatively slow following exposure to 2 M sorbitol whereas Hog1 was rapidly phosphorylated after addition of 1 M NaCl (an equivalent osmolarity to 2 M sorbitol). In *C. albicans*, Hog1 has been shown to be phosphorylated in response to both NaCl and sorbitol [17]. We reasoned that cationic stress is relevant to certain niches during disease progression (e.g., during kidney infection or phagocytosis). Therefore, we chose NaCl as the stressor for further analysis.

We compared two types of oxidative stress: hydrogen peroxide (H_2_O_2_) and tert-butyl alcohol (tBOOH). Both chemicals affected *C. albicans* viability (Fig. 1B) and stimulated Hog1 phosphorylation under the growth conditions examined (Figs. 1C and 1D). Also, both chemicals also affected *C. glabrata* viability (not shown). tBOOH is more stable than H _2_O_2_ and is therefore often used in plate assays for examining oxidative stress resistance [17]. However, our aim was to investigate immediate responses to stresses. Also, H_2_O_2_ is the more physiologically relevant reactive oxygen species as it is generated by phagocytic cells [8,46,47] and therefore it was chosen as the stressor for our subsequent experiments.

We compared two NO donors for the imposition of nitrosative stress: DPTA NONOate and DETA NONOate. These chemicals are reported to have half-lives of about 4 and 20 h, respectively, at 30°C and neutral pH, degrading in first order reactions [48,49]. We used DPTA NON-Oate for our experiments because its shorter half-life was more consistent with the 4-h timescale of our viability experiments, and because this NO donor has been used previously by other groups [13]. The half-life of DPTA NONOate was 130 min under our experimental conditions.

Fourthly, having chosen the stress agents, the next step was to select appropriate stress doses. We chose three doses for each stress (Table 1). The rationale was that this would permit the examination of mechanisms involved in successful adaptation (i.e., to low and medium stresses) or processes that are triggered in the absence of successful adaptation (i.e., to high stresses). Therefore, we selected these doses on the basis of their impact upon *C. glabrata* and *C. albicans* viability and growth as described in the sections below. We also considered the doses that have been used previously [13,14,17,18,32,50]. Some doses differed for *C. glabrata* and *C. albicans* because of their differential stress sensitivities (Table 1). For example, *C. glabrata* is particularly resistant to oxida-tive stress [15].

**Table 1:** Stress doses for *Candida albicans* and *Candida glabrata*

	Doses		
	
	Low	Medium	High
*C. albicans*			
NaCl (M)	0.3	1.0	2.0
H2O2 (mM)	0.4	5.0	20
DPTA-NONOate (mM)	1.25	2.5	7.5
*C. glabrata*			
NaCl (M)	0.1	0.5	2.0
H_2_O_2_ (mM)	1.0	10	100
DPTA-NONOate (mM)	1.25	5.0	7.5

#### Impact of individual stresses upon Candida viability

The impact of each stress condition upon *C. albicans* and *C. glabrata* viability was quantified by measuring the proportion of cells that were propidium iodide positive by FACS analysis. Metabolically active *Candida* cells are able to exclude this dye, whereas necrotic cells are unable to exclude it and become propidium iodide positive [51].

**Fig. 2:**
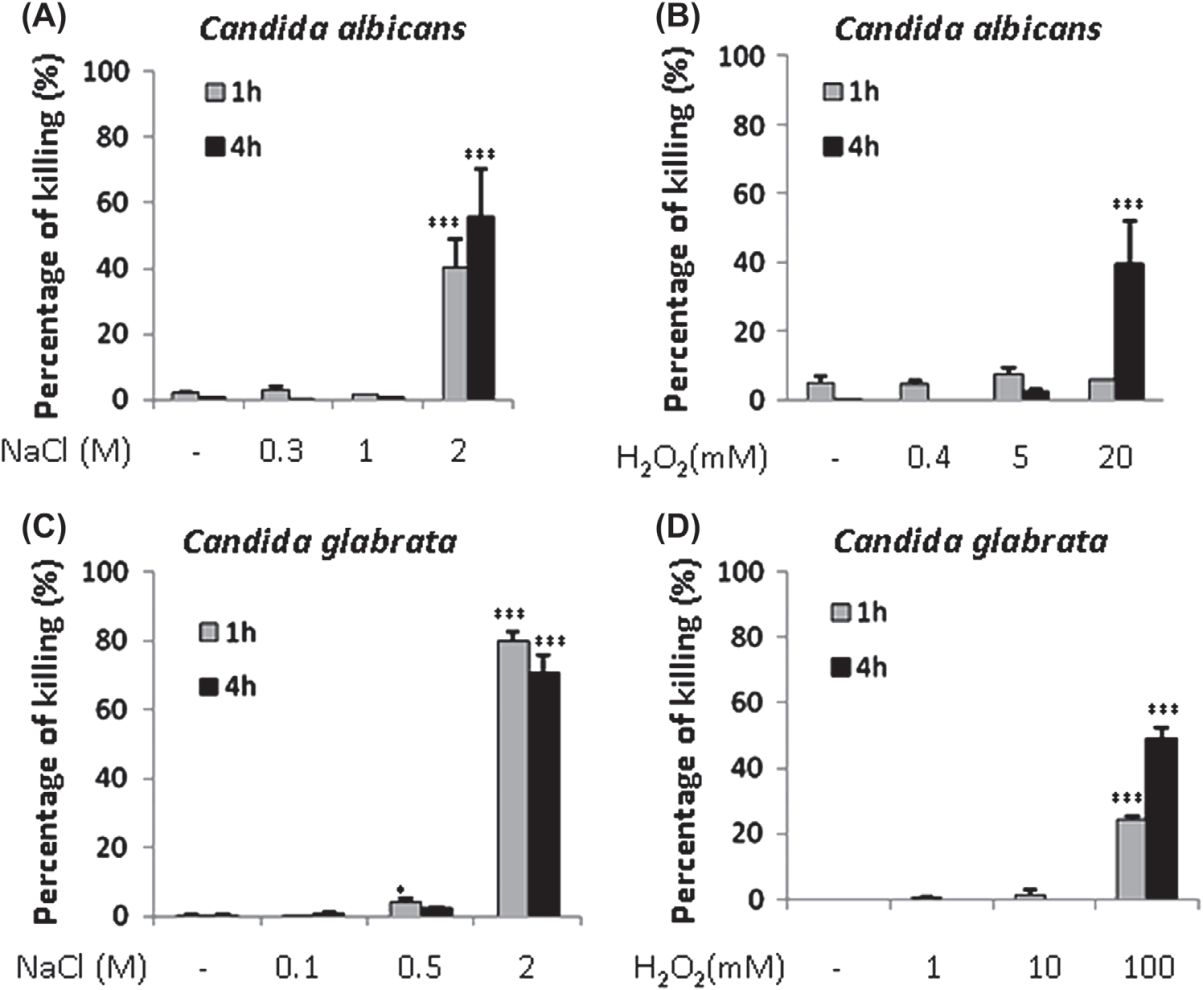
Dose-dependent killing of *Candida albicans* and *Candida glabrata* cells grown in YPDT at 30°C by osmotic and oxidative stresses. Killing was quantified by propidium iodide staining and FACS analysis: blue bars, cell death after 1 h dose of stress; red bars, cell death after 4 h dose of stress. (A) Impact of low, medium and high doses of NaCl upon *C. albicans* viability. (B) Effects of H _2_O_2_ upon *C. albicans* viability. (C) Inf uence of NaCl upon *C. glabrata* viability. (D) Impact of H _2_O_2_ upon *C. glabrata* viability. Values were compared to the no stress controls and significant increases highlighted: ^*^P<0.05; ^**^P<0.01; ^***^P<0.001.

The low and medium doses of NaCl and H_2_O_2_ resulted in low levels of *C. albicans* killing (Figs. 2A and 2B). We chose these doses because previous studies have shown that they are biologically relevant and that gene expression patterns are altered in response to those stress levels [17,18,32,45]. The high dose of NaCl killed over 40% of *C. albicans* cells (Fig. 2A), and the high dose of H _2_O_2_ killed about 40% of cells after 4 h exposure to this stress (Fig. 2B). Similarly *C. glabrata* was relatively resistant to the low and medium doses of NaCl and H _2_O_2_ (Figs. 2C and 2D). *Candida glabrata* was sensitive to the high dose of NaCl, with about 70% of cells being killed under these conditions. Also, *C. glabrata* was sensitive to the high dose of H_2_O_2_, which killed over 40% of cells after 4 h exposure to this stress (Figs. 2C and 2D).

None of the nitrosative stress doses tested (low, medium or high) had an impact on the viability of *C. glabrata* or *C. albicans* (data not shown). This was consistent with reports that nitrosative stress exerts fungistatic rather than fungicidal effects upon *C. albicans* [e.g., 50]. Therefore, the nitrosative stress doses were chosen on the basis of published effects of DPTA-NONOate upon the transcription in *C. albicans* [13,14,45,50].

#### Impact of individual stresses on Candida growth

**Fig. 3:**
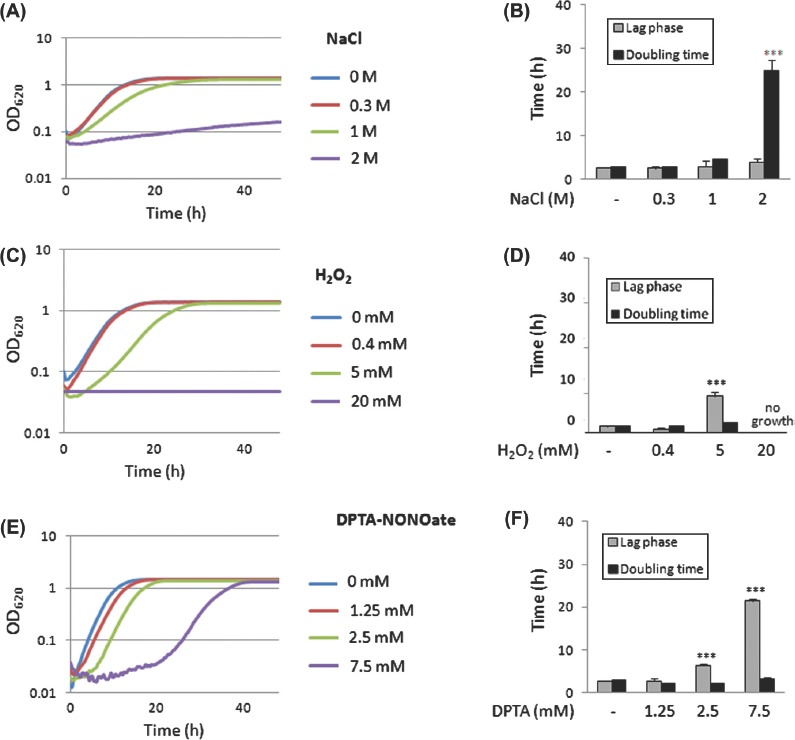
Dose-dependent effects of osmotic, oxidative and nitrosative stresses upon the growth of *Candida albicans* in YPDT at 30°C. The lengths of the lag phase and the doubling time were determined mathematically from growth curves as described in Materials and Methods. (A) Effects of NaCl upon growth. (B) Effects of NaCl upon the length of the lag phase and the doubling time. (C) Effects of H_2_O_2_ upon growth. (D) Effects of H_2_O_2_ upon the length of the lag phase and the doubling time. (E) Effects of DPTA-NONOate upon growth. (F) Effects of DPTA-NONOate upon the length of the lag phase and the doubling time. Values were compared to the no stress controls and significant increases highlighted: ^*^P<0.05; ^**^P<0.01; ^***^P<0.001. This Figure is reproduced in color in the online version of *Medical Mycology*.

Growth curves were generated for both species following exposure to osmotic, oxidative or nitrosative stresses at the low, medium and high doses specified (Table 1). The lengths of the lag phase and doubling times were then calculated mathematically from these growth curves (Fig. 3). We reasoned that the length of the lag phase reflected the time that cells took to adapt to the stress, whereas an increased doubling time probably reflected the increased energetic or metabolic cost of growing in the presence of the stress.

The low dose of NaCl had no significant effects upon the growth of *C. albicans*, whereas the medium dose increased the doubling time slightly without significantly affecting the lag phase, and the high dose dramatically slowed growth over the 48-h period examined (Figs. 3A and 3B). Similarly, the high dose of H _2_O_2_ strongly inhibited the growth of *C. albicans*, whilst the medium dose increased the lag phase (Figs. 3C and 3D). In contrast, the medium and high nitrosative stresses signif cantly increased the length of the lag phase whilst only having slight effects upon the doubling time once growth resumed (Figs. 3E and 3F). Similar studies were performed in *C. glabrata* for the medium dose of each stress (data not shown). The medium osmotic stress had minimal effects on the length of the lag phase or the doubling time of *C. glabrata*. On the other hand, the medium oxidative and nitrosative stresses increased the length of the lag phase.

#### Impact of combinatorial stresses

Having established the common experimental platform and def ned the types and doses of each stress, we were then able to examine the impact of combinatorial stresses upon *C. glabrata* and *C. albicans* on a firm footing. We focussed on cell growth because nitrosative stresses had a minimal impact upon viability, and we started by examining the impact of combinatorial stresses on *Candida* growth using medium stress doses. Growth curves were performed for both species following exposure to the individual stresses and to the different combinations of these stresses, and the lag phases and doubling times quantified mathematically as described above.

**Fig. 4:**
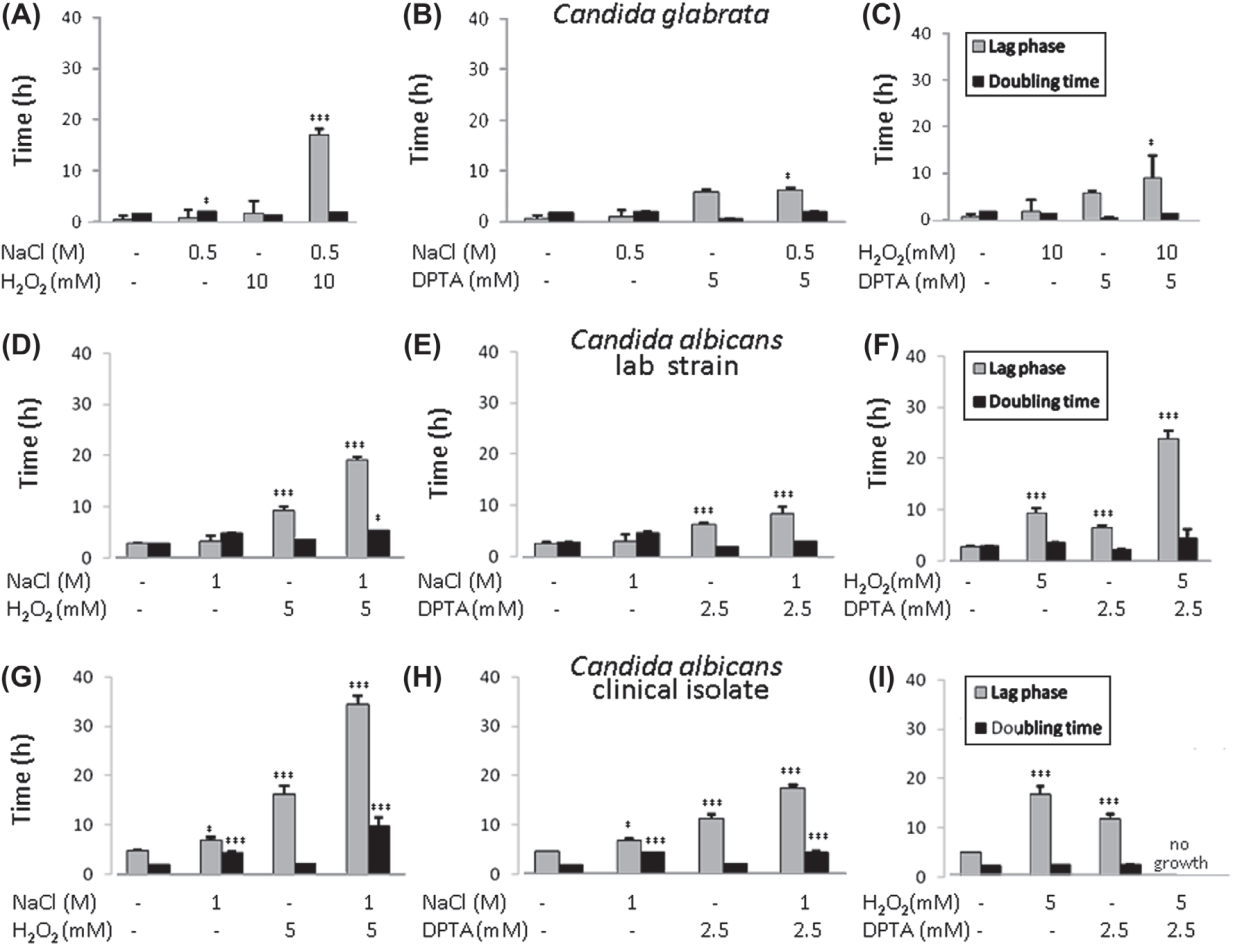
Effects of medium doses of combinatorial stresses upon the growth of cells in YPDT at 30 ° C: (A) (B) and (C) *C. glabrata*; (D) (E) and (F) *C. albicans* strain NGY152; (G) (H) and (I) *C. albicans* isolate SC5314. The lengths of the lag phase and the doubling time were determined mathematically from growth curves as described in *Materials and methods*: gray bars, length of the lag phase; black bars, doubling time. (A) (D) and (G) Comparison of individual and combinatorial osmotic and oxidative stresses. (B) (E) and (H) Comparison of individual and combinatorial osmotic and nitrosative stresses. (C) (F) and (I) Comparison of individual and combinatorial oxidative and nitrosative stresses. Values were compared to the no stress controls and significant increases highlighted: ^*^P<0.05; ^**^P<0.01; ^***^P<0.001.

The medium doses of the individual osmotic, oxidative and nitrosative stresses had minimal effects upon the growth of *C. glabrata* (Figs. 4A–C). However, certain combinations of stress exerted dramatic effects upon the growth. There were significant increases in the length of the lag phase for the combinatorial osmotic plus oxidative stresses and the combinatorial oxidative plus nitrosative stresses (Figs. 4A and 4C) suggesting that *C. glabrata* cells required significantly longer periods to adapt to these combinatorial stresses before they could resume growth, relative to the corresponding individual stresses. In contrast, *C. glabrata* cells were almost as resistant to the combinatorial osmotic plus nitrosative stress as to the single nitrosative stress (Fig. 4B).

A similar picture emerged for *C. albicans* (Figs. 4D–F). The combinatorial osmotic plus oxidative stress had a greater impact upon adaptation time than the corresponding individual stresses (Fig. 4D). Also the combinatorial oxidative plus nitrosative stress resulted in an extended lag phase (Fig. 4F). There was an increase in the length of the lag phase when *C. albicans* were exposed to the combinatorial osmotic plus nitrosative stress compared with the corresponding individual stresses (Fig. 4E). However, this increase was slight, which was consistent with the modest effects of this type of combinatorial stress upon *C. glabrata* cells (Fig. 4B).

The above experiments were performed with a laboratory strain of *C. albicans*. Therefore, to test whether similar combinatorial effects are observed with a clinical isolate we examined the behavior of *C. albicans* SC5314 (Figs. 4G–I). We chose SC5314 because this is the clinical isolate from which NGY152 was derived [37,38]. Both the laboratory strain and clinical isolates displayed similar sensitivities to the individual and combinatorial stresses (Fig. 4).

## Discussion

Our long-term aim is to compare the responses of *C. albicans* and *C. glabrata* to combinatorial stresses and to examine the regulation of these responses in these evolutionarily divergent fungal pathogens. Historically, different experimental practices have evolved for *C. glabrata* and *C. albicans*, and different experimental conditions have evolved for analyses of different types of stressor. Therefore, to achieve our long-term aim, we had to find common experimental ground where we could compare combinations of osmotic, oxidative and nitrosative stresses across both yeasts. The first main aim of this study was to define this common ground.

The experimental platform was first established using buffered rich growth medium at 30°C, and then we chose the types and doses of stressor. Oxidative and nitrosative stresses were chosen because of their physiological relevance to immune defences [9,10]. Osmotic stress was chosen because of its probable relevance in certain host niches and because responses to osmotic stress have been relatively well studied [e.g., 11,52–54]. Regarding the specific stressors and their doses, these were chosen on the basis of their physiological relevance, their chemical properties, their impact on the growth of *C. albicans* and *C. glabrata* (Figs. 1–3), and their use by other groups [13,14,17,23,24,54]. In this way, we def ned low, medium and high doses of NaCl for osmotic stress, of H_2_O_2_ for oxidative stress and of DPTA-NONOate for nitrosative stress (Table 1). Clearly significant differences exist between specific stressors, for example between sorbitol and NaCl (Fig. 1) [53]. Also, the responses of yeasts to stress are dose dependent (Figs. 2–3) [e.g. 17,55]. Nevertheless, we suggest that our choices of stressors and doses provide a solid platform for the initial dissection of combinatorial stress responses in *C. glabrata* and *C. albicans*.

Our next aim was to analyze the effects of individual stresses using this experimental platform, and our data were consistent with previous studies. Nitrosative stresses exerted static rather than cidal effects upon *C. albicans* and *C. glabrata*, whereas oxidative stresses killed these yeasts, and osmotic stresses increased their doubling times (Figs. 2 and 3). Our data also reinforced the view that both

*C. glabrata* and *C. albicans* are relatively resistant to oxi-dative stress [8,12,15]. Indeed, *C. glabrata* is even more resistant to oxidative stress than *C. albicans*, and our doses for this stress were adjusted accordingly (Table 1). The high resistance to oxidative stress could be due to efficient suppression and detoxification of reactive oxygen species by protective enzymes [56]. Apparently, oxidative stress genes in *C. glabrata* are not up-regulated during phagocytosis by macrophages [57]. Therefore, it is not surprising that inactivation of Yap1, Skn7 or Msn2/4 does not affect the viability of *C. glabrata* following contact with macrophages. Only a mutant lacking both Yap1 and Sod1 was killed by macrophages [56]. In *C. albicans*, oxidative stress genes are activated following exposure to neutrophils [45,58]. Also, mutations that inactivate the stress-activated protein kinase Hog1 render *C. albicans* sensitive to a range of stresses including oxidative stress [17,59] and reduce the resistance of cells to killing by neutrophils [60].

Having established the requisite experimental platform, our final aim was to examine the effects of combinatorial stresses. Significantly, we found that both *C. glabrata* and *C. albicans* are more sensitive to certain combinations of stresses and similar results were observed for the *C. albicans* clinical isolate SC5314 (Fig. 4). In particular, the growth of these pathogens was especially sensitive to combinations of oxidative plus osmotic stress or oxidative plus nitrosative stress. In contrast, the growth of *C. glabrata* and *C. albicans* was relatively unaffected by combinatorial osmotic plus nitrosative stresses (Fig. 4). This indicates that the significant impact of combinatorial stresses is specific to certain combinations of stress rather than to general deleterious effects of combining any two stresses.

These observations are highly significant for at least two reasons. First, the potency of specific combinatorial stresses probably contributes to the eff ciency with which neutrophils kill *C. albicans* and *C. glabrata*, thereby suppressing systemic infection in immunocompetent individuals. Second, the significant impact of certain combinatorial stresses upon the growth of these pathogens (relative to the corresponding individual stresses) raises the probability of antagonistic cross-talk between specific stress signalling pathways. Clearly, the largely unexplored topic of combinatorial stress responses promises unexpected and interesting observations that are relevant to infection. Therefore, having established this experimental platform, our aims in the future are to characterize the molecular responses of *C. albicans* and *C. glabrata* to combinatorial stresses, to examine the regulation of these responses and the potential cross-talk between stress-signalling pathways under these conditions, and to establish the significance of combinatorial stresses *in vivo*. We note that this experimental platform provides a strong basis to examine other types of clinically relevant combinatorial stress, for example the simultaneous exposure to thermal stress plus antifungal drugs [61]. We predict that responses to certain types of combinatorial stress may be highly relevant to fungus-host interactions during disease establishment and progression as well as during therapeutic intervention. We also predict that additional physiologically relevant variables *in vivo* such as dynamic changes in ambient pH, nutrient availability and thermal fluctuations are likely to influence these combinatorial effects in interesting and unexpected ways.
